# Nitrate Relationships between Stream Baseflow, Well Water, and Land Use in the Tomorrow-Waupaca Watershed

**DOI:** 10.1100/tsw.2001.294

**Published:** 2001-10-23

**Authors:** Henry Lin, Rebecca Cook, Byron Shaw

**Affiliations:** Department of Crop and Soil Sciences, Pennsylvania State University, University Park 16802, USA

**Keywords:** nitrate leaching, ground water quality, stream baseflow, watershed monitoring

## Abstract

We examined the use of stream baseflow water quality as a representative measure of mean ground water quality in the Tomorrow-Waupaca Watershed in central Wisconsin and the relationship between agricultural land use and watershed water quality. From 1997 to 1999, 38 stream sites were sampled for nitrate during winter and summer baseflow conditions. Some sites have been sampled during winter baseflow conditions since 1994. The land area contributing ground water to each stream sampling site was delineated, resulting in 38 sub-basins. In addition, over 3500 test results from private wells in the watershed were compiled and mapped using a Geographic Information System (GIS). Nitrate concentrations in stream baseflow and well waters were found to have strong positive correlation in the sub-basins of second order or higher. This indicates that stream baseflow may be valid for monitoring mean ground water quality in watersheds predominantly fed by ground water, where much of the stream nitrate is believed to originate from ground water. Analysis of seasonal variation in the stream data showed that winter nitrate concentrations were higher than summer concentrations, implying that winter stream monitoring may be more critical for the assessment of overall ground water quality in the watershed. We also found that, as the amount of agricultural land increased in each sub-basin, average nitrate concentrations in the well and stream waters also increased, suggesting a connection between agricultural land use and nitrate contamination of water resources in the watershed.

### INTRODUCTION

To protect water resources from nonpoint sources (NPS) pollution, a watershed-based approach is needed to understand the relations between water quality and land use. Agriculture is believed to be one of the larger contributors of NPS pollutants to surface and ground waters worldwide. Up until the last few decades, the assumption had been that crops would take up fertilizers, and that soils would adsorb or break pesticides down into harmless byproducts. As early as the 1970s, results from numerous studies showed that fertilizers and pesticides were not being taken up or neutralized before reaching ground water[1,2,3,4,5], and often grain crops barely remove more than 50% of nitrogen fertilizer input[6]. In addition, the advent of irrigated vegetable production on sandy soils, as is the case in central Wisconsin, results in nitrate and other agricultural chemicals being quickly flushed through the coarse soils to the underlying ground water[7,8]. Data from various studies for the last 25 years have illustrated the rapid rate at which nitrate and pesticides may leach through these coarse soils[1,9].

Nitrate from fertilizers and animal wastes is perhaps the most pervasive type of ground water contamination in Wisconsin and elsewhere. Because of poor ground water quality, which also threatens surface water quality, the Tomorrow-Waupaca Watershed (TWW) in central Wisconsin (Fig. 1) was designated as a “priority watershed” by the Wisconsin Department of Natural Resources (WDNR)[10]. Many wells in the watershed have nitrate-N concentration over 10 mg/l (Fig. 1). The WDNR has spent roughly $17 million annually on Priority Watershed Programs statewide in an effort to improve Wisconsin’s water quality[11]. This program targets watersheds with the worst pollution problems and attempts to find ways to upgrade the water quality largely through the implementation of best management practices (BMPs), which range from installing animal waste containment facilities to nutrient management and urban storm water systems. These and related practices have been determined to be the most effective controls of NPS pollution that can be realistically implemented at a reasonable cost. Although BMPs have been implemented in various watersheds, the relations between the BMPs and water quality improvement sometimes have been difficult to assess. Results from some studies even shed doubt on the effectiveness of BMPs to protect ground water. For example, data from several central Wisconsin studies indicated that while some BMPs were effective at reducing ground water contamination, BMPs without additional strategies for rural areas would not protect ground water quality[12,13,14]. An Oklahoma study by Storm et al.[15] also suggested inconclusiveness in determining the effectiveness of BMPs to protect ground water from nitrate associated with farming.

Because there are limited approaches available to monitor changes in ground water quality, and networks of monitoring wells are expensive to install and maintain, this study was conducted to evaluate the potential use of stream baseflow water quality as an indicator of mean ground water quality in the TWW. Since well water is much more costly and difficult to sample than stream water (particularly with privately owned wells and the fact that ground water quality change may take years to show up in domestic wells), if stream water can be used to monitor ground water quality, it would then provide a cost-effective way to assess overall watershed water quality and ground water quality change that may occur in response to BMPs. In this study, we investigated nitrate-N relationships between well water and stream baseflow water in the watershed through several years of monitoring. Seasonal variation of nitrate-N in stream baseflow water samples was also examined to help determine the optimal season to sample when using this approach. In addition, nitrate-N concentrations in the stream and well waters were compared to the amount of land in agricultural production within each sub-basin to examine the relationship between agricultural land use and nitrate level in ground and surface waters in the watershed.

### MATERIALS AND METHODS

#### Study Area — The Tomorrow-Waupaca Watershed (TWW)

The watershed under this study was located in the glaciated areas in central Wisconsin with low hills and uneven topography. The headwaters of the Tomorrow River are in northern Portage County. As the river flows south and southeast, it crosses into Waupaca County and becomes the Waupaca River. Glaciofluvial deposits and till result in a mixture of boulder, gravel, sand, silt, and clay. The areas of till are largely unsorted and unstratified materials, whereas the areas of outwash and delta deposits are generally well-sorted. Both areas tend to yield considerable amounts of water to wells. Annual precipitation in the watershed is approximately 76 cm, with about 25 cm going to ground water recharge[16,17]. Most of the remaining 51 cm is lost to evapotranspiration, although a small amount is lost to runoff. Depth to water table in the watershed ranges from zero to approximately 20 m. Bedrock is found from 1 to 107 m below the surface with predominant type of granite[16,17]. There are 80 lakes in the watershed and 16 named tributaries, many of which are predominantly ground water fed. Internal drainage takes place in over half the basin, with surface waters flowing to potholes and small ponds. The high degree of interaction between surface water and ground water in the TWW allows surface water quality to be impacted by the quality of ground water, and vice versa[10].

Soils of the watershed are relatively young and predominantly sand and gravel, ranging from sandy to loamy in texture, and from excessively well-drained to a few areas that are poorly drained. In the majority of the watershed, there is a lack of significant clay content to provide filtering or containment of nutrients and other pollutants[18].

Land use in the watershed is 55% agricultural, 35% forested, 5% wetlands, and 5% developed for approximately 13,000 residents. As of 1998, there were about 145 stockyards/barnyards in the watershed; 80 of these are full-time dairy or beef farmers, and 65 are hobby farms averaging 20 heads each[19]. This agriculture-dominant watershed makes agricultural practices an obvious focal point in the battle to improve local water quality. Of the agricultural land, 82% is in nonirrigated production growing mainly corn and hay, and 18% is irrigated producing potatoes, peas, sweet corn, snap beans, soybeans, and cucumbers. Corn and potato fields and animal wastes are believed to be the major contributors to water contamination in the watershed.

#### Stream and Well Water Quality Database

There were 38 ground water sub-basins delineated within the TWW using ArcView Geographic Information System (GIS) (ESRI Inc., Redlands, CA). The sub-basins were delineated for each stream sampling site based on the ground water contours, estimated ground water recharge rates, and the average baseflow measurements for each site. The mean of baseflow measurements for each site was used to determine how much land should be in each sub-basin in order to contribute the amount of baseflow water measured. An attempt was made to have a minimum of one sampling site at the headwaters and mouth of the major tributaries, as well as along the main river channel and minor tributaries. This resulted in 10 sampling sites on the Tomorrow-Waupaca River and 28 sites on the tributaries (Fig. 1).

Stream water samples were collected from the 38 sites during winter baseflow conditions since 1994. From July 1997 to January 1999, both summer and winter baseflow sampling was conducted. Because of the permeable nature of the soils in the watershed, baseflow conditions were generally reached after 3 days without precipitation or a snow melt event. The water samples were collected in 250-ml polyethylene bottles preserved

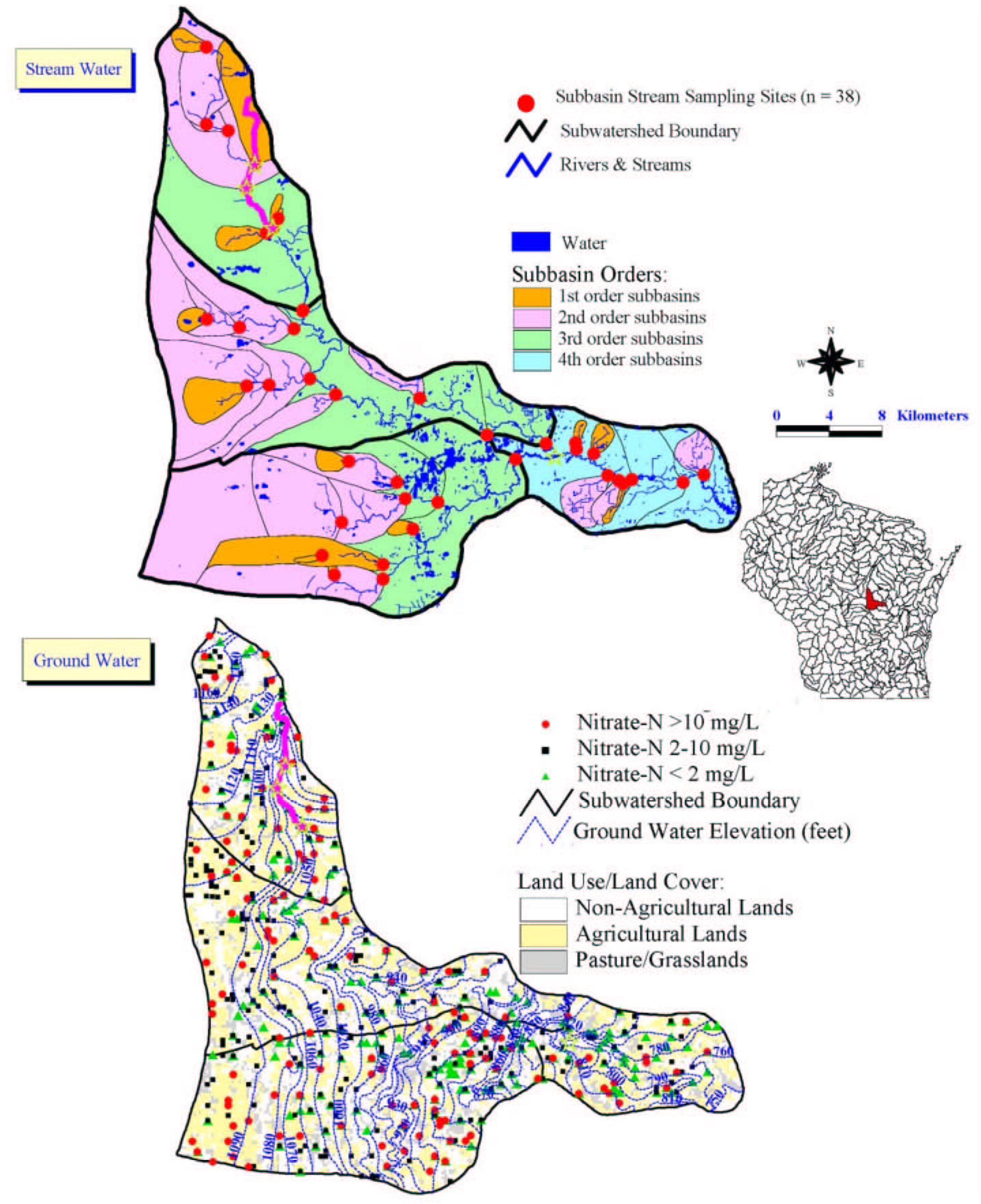

Fig. 1. Stream water (above) and ground water (below) samples collected in this study in the Tomorrow -Waupaca Watershed in central Wisconsin, along with some watershed background information.

**FIGURE 1**. Stream water (above) and ground water (below) samples collected in this study in the TWW in central Wisconsin, along with some watershed background information.

with $H_2SO_4$ and transported to the laboratory on ice. At the same time that the water samples were collected, stream flow measurements were taken with a Marsh McBirney model 2000 flow meter. An attempt was made to collect all samples and flow measurements within a 24-h period for any given sampling event. Each sample was then analyzed for nitrate-N concentration, as well as other water quality parameters not reported in this paper. The analyses were done in the state-certified Environmental Task Force Laboratory at the University of Wisconsin–Stevens Point using approved methods.

An extensive private well water database containing nitrate concentration and other water quality parameters for the watershed was also developed over the years through the Environmental Task Force Laboratory, the Ground Water Retrieval Network of the WDNR, and Portage County Health Laboratory. A database comprising of over 3500 test results from private wells in the TWW were then compiled and mapped using ArcView GIS (Fig. 1). When the well data were mapped and evaluated spatially, it was determined that some areas in the watershed had minimal well water sampling. These areas were targeted for a door-to-door sampling campaign in which free well water analysis was offered in order to obtain data for the area. A total of 100 additional well water samples were so obtained in this project.

### GIS and Statistical Analysis

ArcView GIS was used in this study to spatially evaluate the database and to analyze the collected data. Besides those factors mentioned above, a land use map was also used to determine the percentage of each land use category in the delineated sub-basins. After sorting the data by sub-basin order, parametric and nonparametric statistical methods were used for data analysis using the Statistical Analysis System (SAS Institute Inc., Cary, NC). Since most of the data were not normally distributed, nonparametric statistics was deemed more appropriate[20]. Correlations between the stream baseflow water chemistry and the well water chemistry were run with the Spearman's rho test, a test that measures monotonic relationships based on ranks and resists the effects of outliers. Because there were only a few third- and fourth-order sub-basins in this study, the sample size was too small to use the Spearman's rho test; thus the Pearson correlation test was used instead. The significance of the seasonal variation in chemical concentrations in the stream water was analyzed with a paired t-test because of the small data set.

## RESULTS AND DISCUSSION

### Nitrate Relationship between Stream Baseflow and Well Water

The correlation between the average nitrate-N concentrations in the well water and the winter baseflow water was noticeable in the sub-basins greater than the first order (Fig. 2a). We believe that the poor nitrate-N correlation for the first-order stream sub-basins was related to small sub-basin size and significant variability at local level (Figs. 2a and 3b). Because the first-order sub-basins were small (0.15 to 14.3 $km^2$), it was difficult to collect a representative number of private well water samples. Two of the first-order sub-basins did not have any well water data, as there were no wells within their boundaries from which to collect ground water. In addition, when working with small sub-basin areas, any error in the delineation of the sub-basin boundaries could be significant. Another factor might be related to the fact that the drinking-water wells were much deeper than the water that contributed to stream flow. Thus, well water may not have the same nitrate-N concentration as streams, especially the first-order streams.

Nevertheless, the apparent positive correlation for larger sub-basins (second order and above) implies that there is a significant relationship between well water quality and stream water quality during baseflow conditions (Fig. 2a). Since the TWW is predominantly ground water fed, much of the stream nitrate in the watershed originates from the ground water. Although the stream baseflow would not have the same levels of nitrate as the well water because of the travel time that ground water takes to discharge to stream water and possible biological and chemical interference that might occur during the travel, a regression equation (such as those in Fig. 2a) may be established for each unique watershed or its sub-basins to determine the relationship between stream baseflow and well water chemistry. Once such equation is established, the test results from stream baseflow samples could then be used to estimate mean ground water quality. It would seem that monitoring changes in stream baseflow water quality could reflect changes in mean ground water quality in sub-basins of second order and perhaps higher in the watershed. Note, though, the number of third- and fourth-order sub-basins in this study was small. Thus, the validity of such relationship in sub-basins greater than the second order would need further examination. In particular, there were only two fourth-order stream sub-basins in this study, thus its regression equation would not be meaningful and therefore is not shown in Fig. 2.

### Seasonal Variation in Stream Baseflow Nitrate Concentration

The winter and summer baseflow nitrate-N concentrations were compared using the data from the summer of 1997 through the winter of 1999, the only 2 years for which we had both summer and winter data. For sub-basins greater than the first order, winter baseflow had greater average nitrate-N concentrations than the summer baseflow, with a significant level over 99% (Fig. 3a). The reason is likely related to the fact that some nitrate might have been taken up by vegetation in the streams during the summer and/or stronger denitrification might have occurred during the ground water discharge to the streams in the summer. The difference by season may be also related to residence time for water, as crops are fertilized in spring and summer and nitrate may flow to ground water in later summer and to streams in winter. Thus, it would be more desirable to use winter baseflow to monitor ground water quality in the watershed.

Variability in the winter baseflow nitrate levels using data from 1994 to 1999 showed that the first-order streams had the widest variation among the different orders of streams (Fig. 3b). Such wide variability occurred mainly in agriculture-dominated sub-basins, and those with forested lands had small standard deviation. This is a proof that nitrate concentrations in streams in winter were associated with previous nitrogen use on croplands. The standard deviation in sub-basins of second order and above was considerably smaller (Fig. 3b). This reveals a possible decreasing trend of stream nitrate-N concentration and its reduced variation with increasing sub-basin order. This may be explained by local land use and soils impacts on water quality being the most variable in the first-order sub-basins and then that being gradually "homogenized" down stream (i.e., increasing sub-basin order).

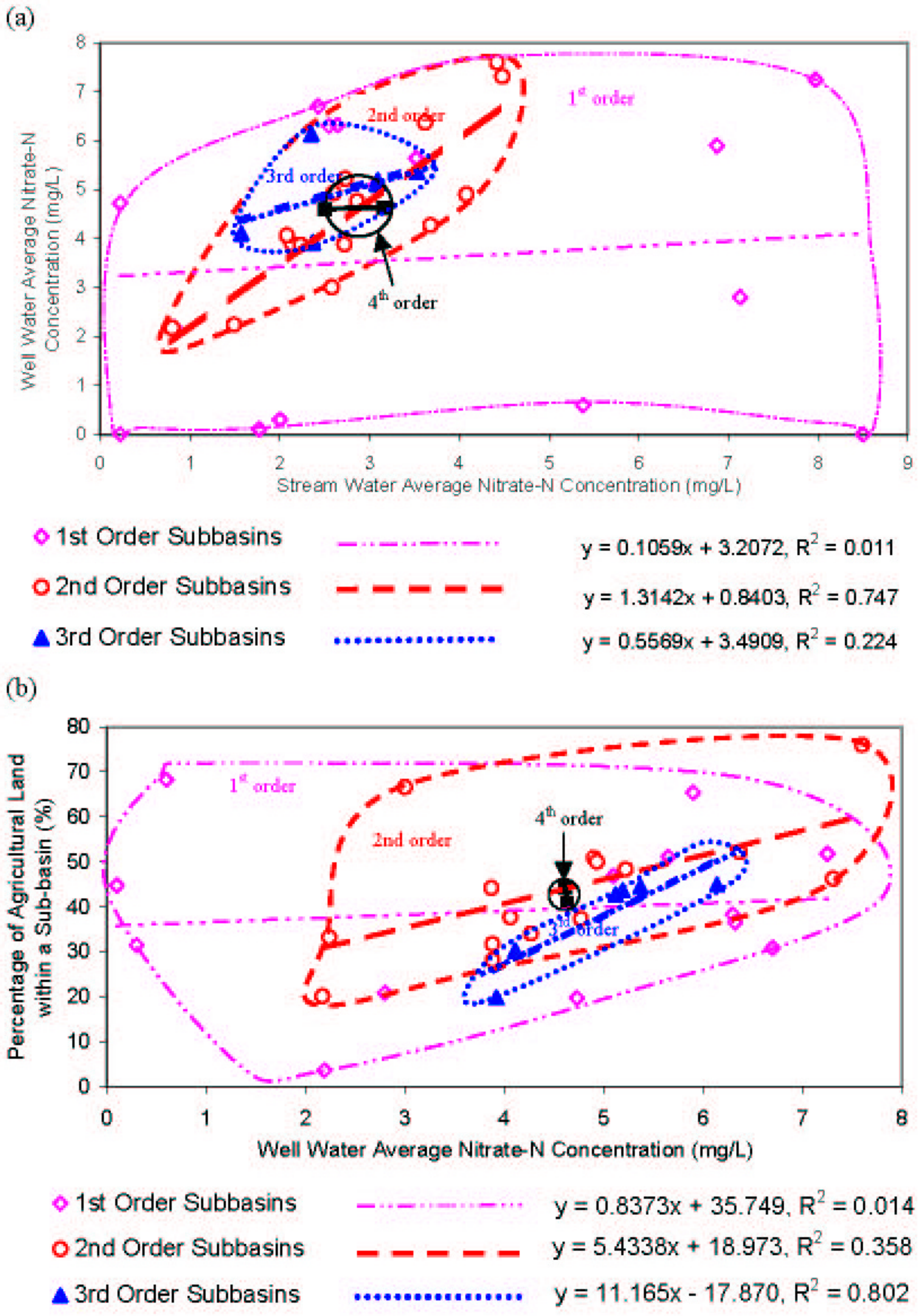

**FIGURE 2**. (a) Nitrate-N relationship between stream baseflow and well water in the first- to fourth-order stream sub-basins in the TWW; (b) well water nitrate-N concentration in relation to the percentage of agricultural land in the first- to fourth-order stream sub-basins in the TWW.

### Nitrate and Agricultural Land Use

The percentage of land in agricultural production within each sub-basin was examined to see if there was a connection with nitrate-N averages in the private wells and baseflow samples. There did not seem to be a direct correlation between average nitrate-N concentrations in wells or baseflow samples and the amount of land in irrigated agricultural production in any orders of the sub-basins. However, when irrigated and nonirrigated land was combined, the average nitrate-N concentrations in the sub-basin wells and baseflow samples increased as the amount of agricultural land increased, except for the first-order sub-basins (Fig. 2b). Also, the number of the fourth-order sub-basins was only two, thus its validity could not be determined. The trend of increasing nitrate concentration in ground and surface waters with increasing agricultural land in the second- and third-order sub-basins appears to confirm that the majority of the nitrate contamination in the watershed has come from the agricultural fields,

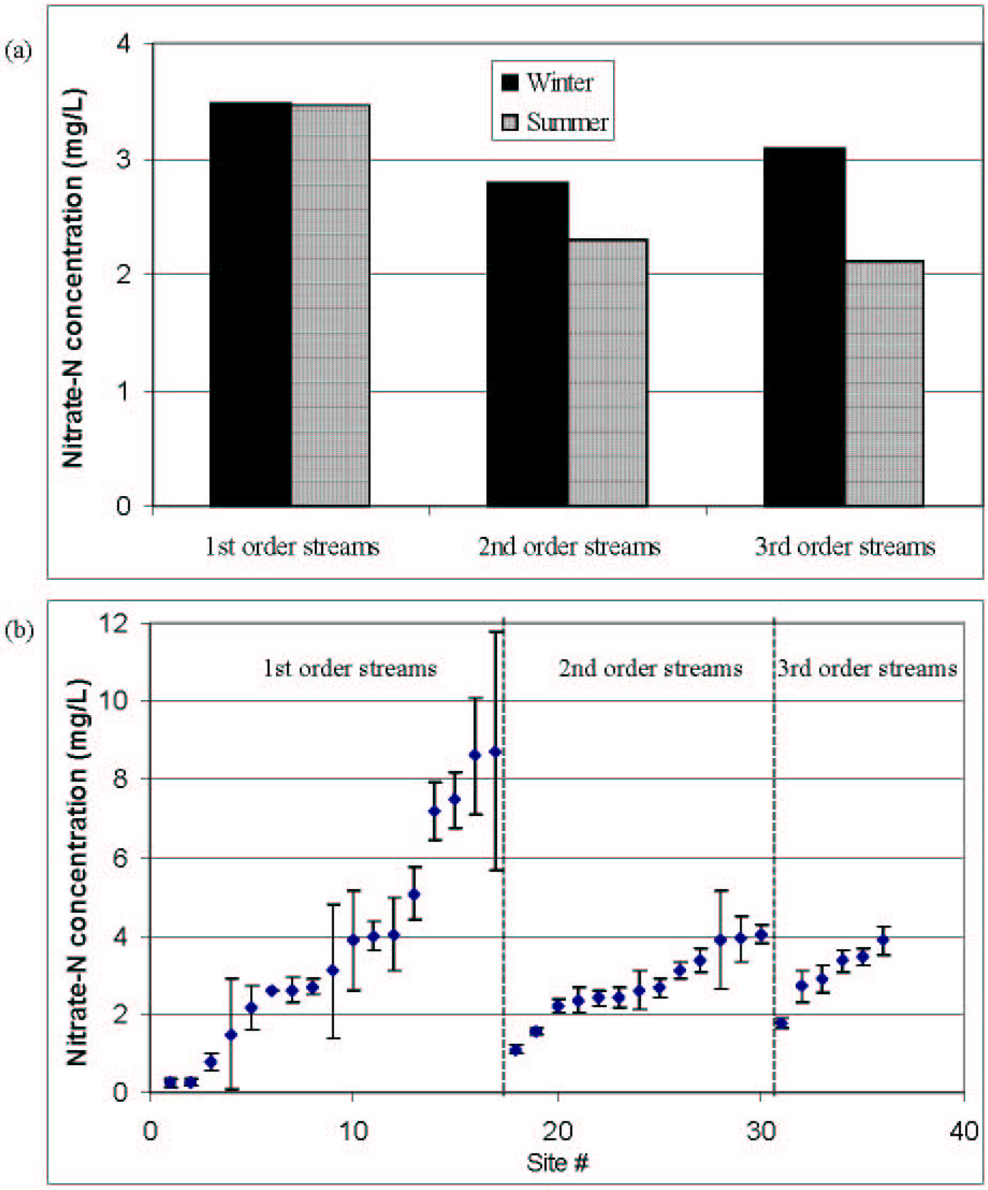

**FIGURE 3**. (a) Comparison of average nitrate-N concentrations between summer and winter baseflow waters sampled from 1997 to 1999 in different orders of streams; and (b) mean and standard deviation of winter baseflow nitrate-N concentrations in the first, second, and third order of streams from 1994 to 1999 in the TWW.

where high crop yield is the key consideration in nitrogen application rates despite environmental ramifications. This result is consistent with considerable literatures that have suggested a close tie between agricultural land use and water resources contamination. For example, Jordan et al.[21] investigated 27 watersheds having differing proportions of cropland in the Piedmont and Coastal Plan provinces of the Chesapeake Bay drainage and found that the percentage of cropland could explain 45 to 51% of variance in stream flow-weighted mean nitrate concentration. A study by Burkart and Kolpin[22] showed that wells located in areas with at least 25% corn or soybeans had significantly higher nitrate concentrations than wells in less densely cropped areas. Hill[23] found ground water nitrate-N concentration under potato fields to be >10 mg/l in 41% of the ground water samples taken, whereas beneath forests or pastures the concentrations were <1 mg/l.

### CONCLUSIONS

Stream baseflow could be used to monitor mean ground water quality in the TWW in sub-basins greater than the first order. Because of seasonal variation, winter baseflow monitoring would be more critical for the assessment of ground water quality in the watershed. As the amount of agricultural land increased in sub-basins of second order or higher, average nitrate concentrations in the well and stream waters also increased, suggesting a likely connection between agricultural land use and water nitrate contamination in the watershed.

### ACKNOWLEDGMENTS

Funding for this study was provided in part by the Wisconsin Department of Natural Resources, the University of Wisconsin Water Resources Institute, and the USDA-NRI grant #2001-35102-10044.

| | | |
|---|---|---|
| Received: | July | 23, 2001 |
| Revised: | September | 24, 2001 |
| Accepted: | October | 9, 2001 |
| Published: | October | 23, 2001 |

### BIOSKETCH

**Henry Lin** is an Assistant Professor of Hydropedology/Soil Hydrology in the Department of Crop and Soil Sciences at the Pennsylvania State University, University Park, PA. His research and teaching interests include two aspects: one is fundamental study of flow and transport processes in the vadose zone and watershed, with an emphasis on bridging disciplines, scales, and data; and the other is the application of geospatial technologies, environmental modeling, and artificial intelligence in soil and water sciences, with a particular interest in developing Online Advanced Spatial Information Systems (OASIS). Dr. Lin holds a B.S. in Soil Science and Agricultural Chemistry from Fujian Agricultural University, China (1985), a M.S. in Pedology/Soil Geography from Nanjing Institute of Soil Science, the Chinese Academy of Sciences (1988), and a Ph.D. in Soil Physics and Pedology from Texas A&M University (1995).
